# Dermoscopic Demonstration of Darier Sign

**DOI:** 10.5826/dpc.1101a114

**Published:** 2021-01-29

**Authors:** Sweta Mukherjee, Shekhar Neema, Preema Sinha, Sunmeet Sandhu, A.W. Kashif, S. Radhakrishnan

**Affiliations:** 1 Department of Pediatrics, Command Hospital, Pune, India; 2 Department of Dermatology, Armed Forces Medical College, Pune, India; 3 Department of Pathology, Armed Forces Medical College, Pune, India

**Keywords:** Darier sign, mastocytosis, dermoscopy, dermatoscopy

## Case Presentation

**Figure 1:**
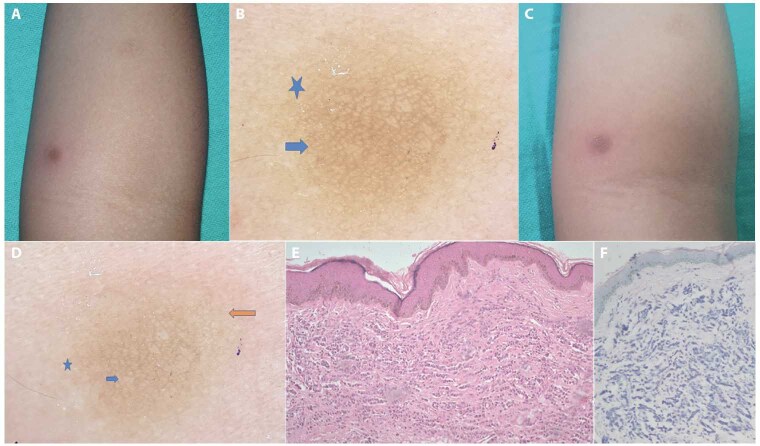
(A) Solitary plaque measuring 1 × 1 cm over the inner aspect of forearm. (B) Polarized dermoscopy shows pigment network (blue arrow) on a yellow background (blue star) (DermLite DL4, ×10). (C) Same plaque after eliciting Darier sign: plaque has become more well-defined, surface appears like orange-peel, and surrounding skin is erythematous. (D) Polarized dermoscopy after eliciting Darier sign shows decrease in intensity of yellow hue (blue star), decreased density of pigment network (blue arrow), and surrounding erythema (orange erythema). The pigment network is broader compared to Figure 1C due to edema in papillary dermis (DermLite DL4, ×10). (E) Histopathology shows hyperpigmentation of basal layer (H&E, ×4) and (F) presence of mast cells in the papillary and reticular dermis (H&E, ×4; toluidine blue stain ×10).

A 9-month-old child presented with a 3-month history of a solitary hyperpigmented plaque having a leathery surface and a peau d’orange appearance located on the inner aspect of left forearm (Figure 1A), dermoscopically characterized by a pigment network on a yellow background (Figure 1B). Urtication of the lesion upon scratching (Darier sign) was evident (Figure 1C) and decreased pigment network density and yellow hue intensity on dermoscopy. (Figure 1D). Histopathology was consistent with mastocytoma (Figures 1, E and F).

## Teaching Point

Darier sign is only seen in 50% of cases of mastocytomas on clinical grounds [1]. Dermoscopic features of mastocytosis include light brown blots, pigment network, reticular vascular pattern and yellow-orange blots [2]. Pigment network and yellow-orange blots histopathologically correspond to basal layer hyperpigmentation and dense mast cell infiltrate, respectively. Eliciting Darier sign points to degranulation of mast cells which leads to erythema and edema that in turn cause decrease in a yellowish hue and pigment network intensity along with appearance of peripheral erythema on dermoscopy.
